# Phylogeographical and population genetics of *Polyspora* sweet in China provides insights into its phylogenetic evolution and subtropical dispersal

**DOI:** 10.1186/s12870-024-04783-5

**Published:** 2024-02-06

**Authors:** Zhifeng Fan, Can Gao, Lifang Lin

**Affiliations:** 1 City College, Kunming University of Science and Technology, Kunming, 650093 China; 2 College of Landscape Architecture and Horticulture Sciences, Southwest Forestry University, Kunming, 650224 China; 3 Hot Spring Sub-district Office, Anning Municipal People’s Government, Kunming, 650300 China

**Keywords:** *Polyspora*, Origin and evolution, Phylogeography, Genetic structure, Chloroplast haplotype

## Abstract

**Background:**

Geological movements and climatic fluctuations stand as pivotal catalysts driving speciation and phylogenetic evolution. The genus *Polyspora* Sweet (Theaceae), prominently found across the Malay Archipelagos and Indochina Peninsula in tropical Asia, exhibits its northernmost distribution in China. In this study, we investigated the evolutionary and biogeographical history of the genus *Polyspora* in China, shedding light on the mechanisms by which these species respond to ancient geological and climatic fluctuations.

**Methods:**

Phylogenetic relationships of 32 representative species of Theaceae were reconstructed based on the chloroplast genome and ribosome 18-26 S rRNA datasets. Species divergence time was estimated using molecular clock and five fossil calibration. The phylogeography and population genetics in 379 individuals from 32 populations of eight species were analyzed using chloroplast gene sequences (*trn*H-*psb*A, *rpo*B-*trn*C and *pet*N-*psb*M), revealing the glacial refugia of each species, and exploring the causes of the phylogeographic patterns.

**Results:**

We found that Chinese *Polyspora* species diverged in the middle Miocene, showing a tropical-subtropical divergence order. A total of 52 haplotypes were identified by the combined chloroplast sequences. Chinese *Polyspora* exhibited a distinct phylogeographical structure, which could be divided into two clades and eight genealogical subdivisions. The divergence between the two clades occurred approximately 20.67 Ma. Analysis of molecular variance revealed that the genetic variation mainly occurred between species (77.91%). At the species level, *Polyspora axillaris* consists of three lineages, while *P. speciosa* had two lineages. The major lineages of Chinese *Polyspora* diverged between 12 and 15 Ma during the middle to late Miocene. The peak period of haplotype differentiation in each species occurred around the transition from the last interglacial to the last glacial period, approximately 6 Ma ago.

**Conclusion:**

The primary geographical distribution pattern of Chinese *Polyspora* was established prior to the last glacial maximum, and the population historical dynamics were relatively stable. The geological and climatic turbulence during the Quaternary glacial period had minimal impact on the distribution pattern of the genus. The genus coped with Quaternary climate turbulence by glacial in situ survival in multiple refuges. The Sino-Vietnam border and Nanling corridor might be the genetic mixing center of *Polyspora*.

**Supplementary Information:**

The online version contains supplementary material available at 10.1186/s12870-024-04783-5.

## Background

Species differentiation and lineage evolution are jointly influenced by geological movement and climate change [1]. Since the collision between Indian plate and Eurasian plate, the Himalayas have experienced continuous and rapid uplift in the Miocene. With the establishment and strengthening of the East Asian summer monsoon, the arid climate region in the southern subtropical zone of China disappeared and transformed into a warm and humid monsoon climate [2]. The global temperature showed an general downward trend after the Middle Miocene Climatic Optimum (MMCO, 15–17 Ma) period, with a large uplift of the Himalayas in the late Miocene (7–9 Ma) [3]. Although the geological movement had slowed down since the Pleistocene, the temperature was still falling, causing numerous glacial-interglacial fluctuations. These geological and climatic changes have profoundly influenced the formation and evolution of species lineages. However, previous studies have primarily focused on the effects of Quaternary glacial climate fluctuations on species divergence and distribution changes, rarely explored the impacts of long-term geological and climatic changes on the divergence, speciation and geographic lineage evolution of phytogroup, especially during the Oligocene and Miocene epochs.

*Polyspora* Sweet belongs to tribe Theeae of Theaceae, species within the genus are evergreen tree or shrub which blooming in winter. They are excellent landscaping tree, mountain afforestation tree, and timber tree. The genus consists of 47 accepted species, mainly distributed in Malay Archipelago and Indochina Peninsula in tropical Asia. *Polyspora* has an ancient origin and has experienced the whole Quaternary climate change process. It is widely distributed and covered most of the subtropical regions of China [4]. China is on the northern edge of the distribution of *Polyspora*, which has both tropical components, Chinese *Polyspora* species is also a representative taxon of tropical-subtropical transition. Therefore, the study on phylogeography of Chinese *Polyspora* can provide a typical case for the impact of paleogeology and climate change on plant population dynamics, and also provide important data for exploring species exchange between tropical and subtropical regions.

**Fig. 1:**
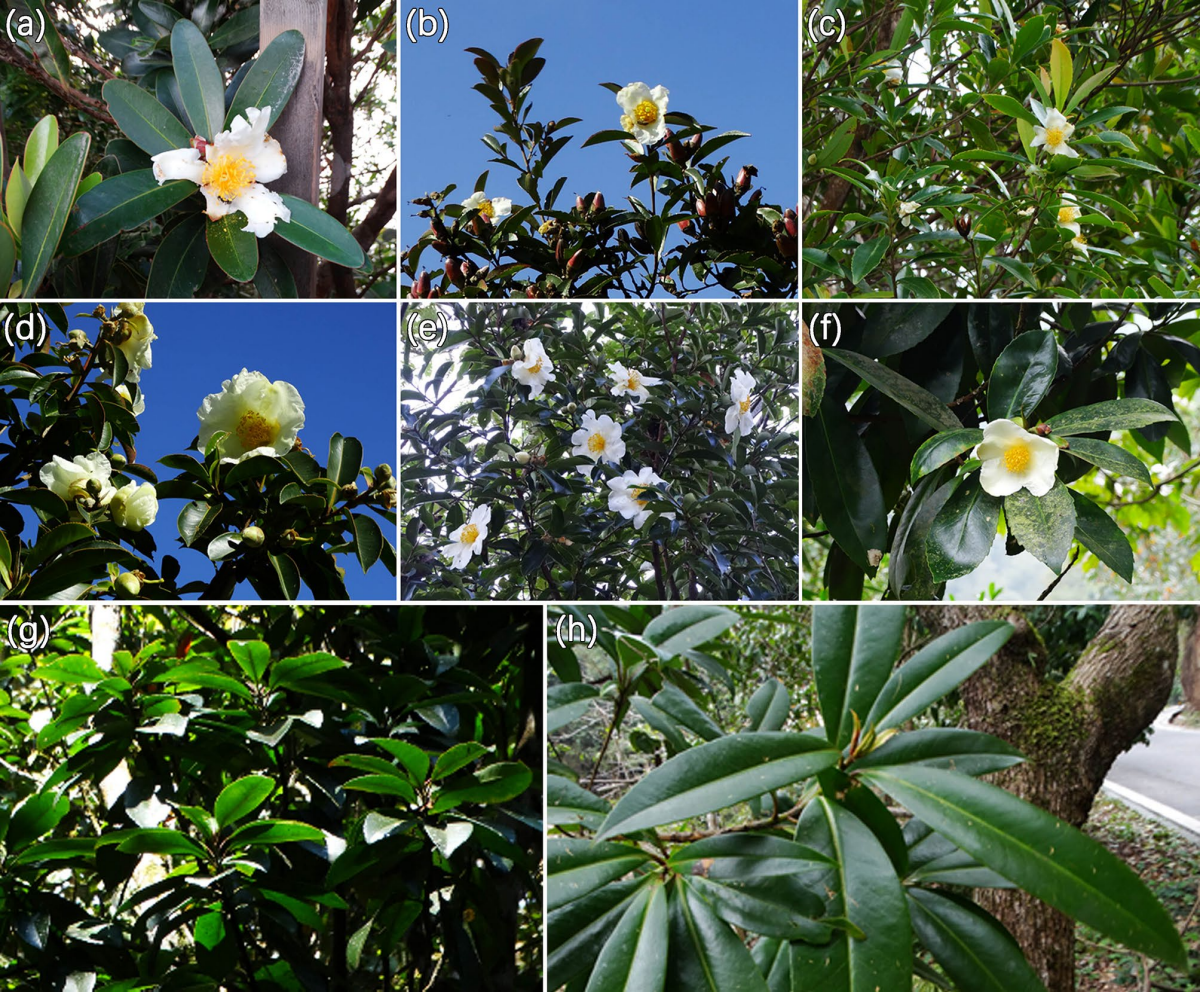
Photos of Chinese *Polyspora* species. **(a)**  *Polyspora axillaris* (Roxb. ex Ker Gawl.) Sweet; **(b)**  *P. chrysandra* (Cowan) Hu ex B.M.Barthol. & T.L.Ming; **(c)**  *P. hainanensis* (Hung T.Chang) C.X.Ye ex S.X.Yang; **(d)**  *P. longicarpa* (Hung T.Chang) C.X.Ye ex B.M.Barthol. & T.L.Ming; **(e)**  *P. tiantangensis* (L.L.Deng & G.S.Fan) S.X.Yang; **(f)**  *P. speciosa* (Kochs) B.M.Barthol. & T.L.Ming; **(g)** *P. tonkinensis* (Pit.) S.X.Yang; **(h)** *P. kwangsiensis* (Hung T.Chang) C.X.Ye ex S.X.Yang

According to Yang Shixiong’s [5] taxonomic treatment of *Polyspora*, there are 8 species of *Polyspora* in China (including 2 endemic species, Fig. 1), namely: *P. axillaris* (Roxb. ex Ker Gawl.) Sweet, *P. speciosa* (Kochs) B.M.Barthol. & T.L.Ming, *P. chrysandra* (Cowan) Hu ex B.M.Barthol. & T.L.Ming, *P. longicarpa* (Hung T.Chang) C.X.Ye ex B.M.Barthol. & T.L.Ming, *P. hainanensis* (Hung T.Chang) C.X.Ye ex S.X.Yang (endemic), *P. tiantangensis* (L.L.Deng & G.S.Fan) S.X.Yang (endemic), *P. tonkinensis* (Pit.) S.X.Yang and *P. kwangsiensis* (Hung T.Chang) C.X.Ye ex S.X.Yang, which are mainly distributed in the southwestern and southern China [4]. However, when Flora of China (FOC) [6] revised the genus *Polyspora*, *P. kwangsiensis* was merged into *P. speciosa*, and *P. tonkinensis* was merged into *P. axillaris*, the number of Chinese *Polyspora* species was changed to 6, while *P. tiantangensis* was considered as a questionable species. Some other scholars hold different views on the taxonomic treatment [7].

To accurately infer the phylogenetic relationship of Chinese *Polyspora*, we utilized the chloroplast (cp.) genome and ribosome 18-26 S rRNA dataset to reconstruct the phylogenetic relationships within Theaceae. Five fossil calibration points were used to estimate the species divergence time of *Polyspora* by molecular clock. On this basis, three chloroplast fragments (*trn*H-*psb*A, *rpo*B-*trn*C, and *pet*N-*psb*M) were employed to examine the phylogeography and population genetics of Chinese *Polyspora*. We conducted analyses pertaining to genetic diversity, population genetic structure and historical dynamic. By summarizing the species divergence and genealogical evolutionary history of the genus *Polyspora*, we aim to: (1) elucidate the phylogenetic relationships of the genus *Polyspora*, as well as the major taxa within Theaceae, and to furnish reliable molecular information for the taxonomic revision of Chinese *Polyspora*; (2) reveal lineage structures and glacial refugia, estimate lineage divergence times, and investigate the factors contributing to the formation of phylogeographic patterns.

## Results

### Molecular phylogenetic analysis

**Fig. 2:**
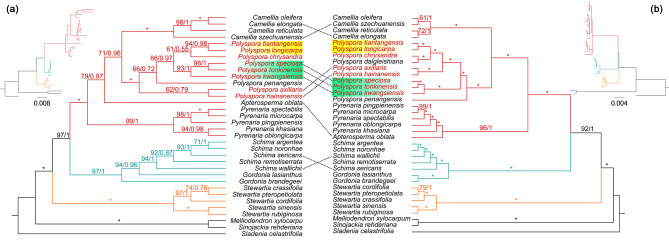
Comparison of ribosomal 18-26 S rRNA and chloroplast genome phylogenetic tree. **(a)** ribosome tree; **(b)** chloroplast genome tree. The numbers preceding the slashes on the nodes indicate the support rates from the ML tree, while the numbers following the slashes represent the posterior probability values acquired from the BI tree. Asterisks denote nodes with maximum support, signified by 100% ML support rates and 1.00 BI posterior probability values

The results of Maximum likelihood (ML) and Bayesian inference (BI) trees based on chloroplast genomes (Fig. 2b, Figure S1) showed that strong support for the majority of branches in the cpDNA phylogenetic tree, and the three clades corresponded to three tribes of Theaceae. The four genera of *Polyspora* Sweet, *Camellia* L., *Pyrenaria* Blume, and *Apterosperma* Hung T. Chang constituted tribe Theeae. *Gordonia* Ellis (native to North America) and *Schima* Reinw. ex Blume formed a sister clade, and the two genera formed tribe Gordonieae. Ten species of genus *Polyspora* formed a monophyletic lineage, with an internal branch support/posterior rate of 100%/1.00. *Polyspora penangensis* (Ridl.) Niissalo & L.M.Choo from Malaysia was located at the base of the genus. *P. longicarpa* and *P. tiantangensis*, *P. chrysandra* and *P. dalgleishiana* (Craib) Orel, Peter G.Wilson, Curry & Luu (from Vietnam), *P. axillaris* and *P. hainanensis*, *P. speciosa*, *P. tonkinensis* and *P. kwangsiensis* formed four clades within the genus, respectively. Except for *P. speciosa* clade, the other three clades had the same or adjacent geographical distribution.

The ML/BI phylogenetic tree of Theaceae constructed using ribosomal 18-26 S rRNA from 34 species (Fig. 2a, Figure S2), showed that *P. longicarpa* and *P. tiantangensis*, *P. speciosa*, *P. tonkinensis* and *P. kwangsiensis* also formed highly supported monophylets. Combined with cpDNA phylogeny, little nuclear-plastid conflicts existed, primarily manifested within the clades of *P. axillaris* and *P. speciosa*. The chloroplast phylogenetic tree suggested that *P. speciosa* was diverged early, whereas the ribosomal phylogenetic tree showed that *P. axillaris* was the early divergent lineage.

### Estimation of differentiation time

**Fig. 3:**
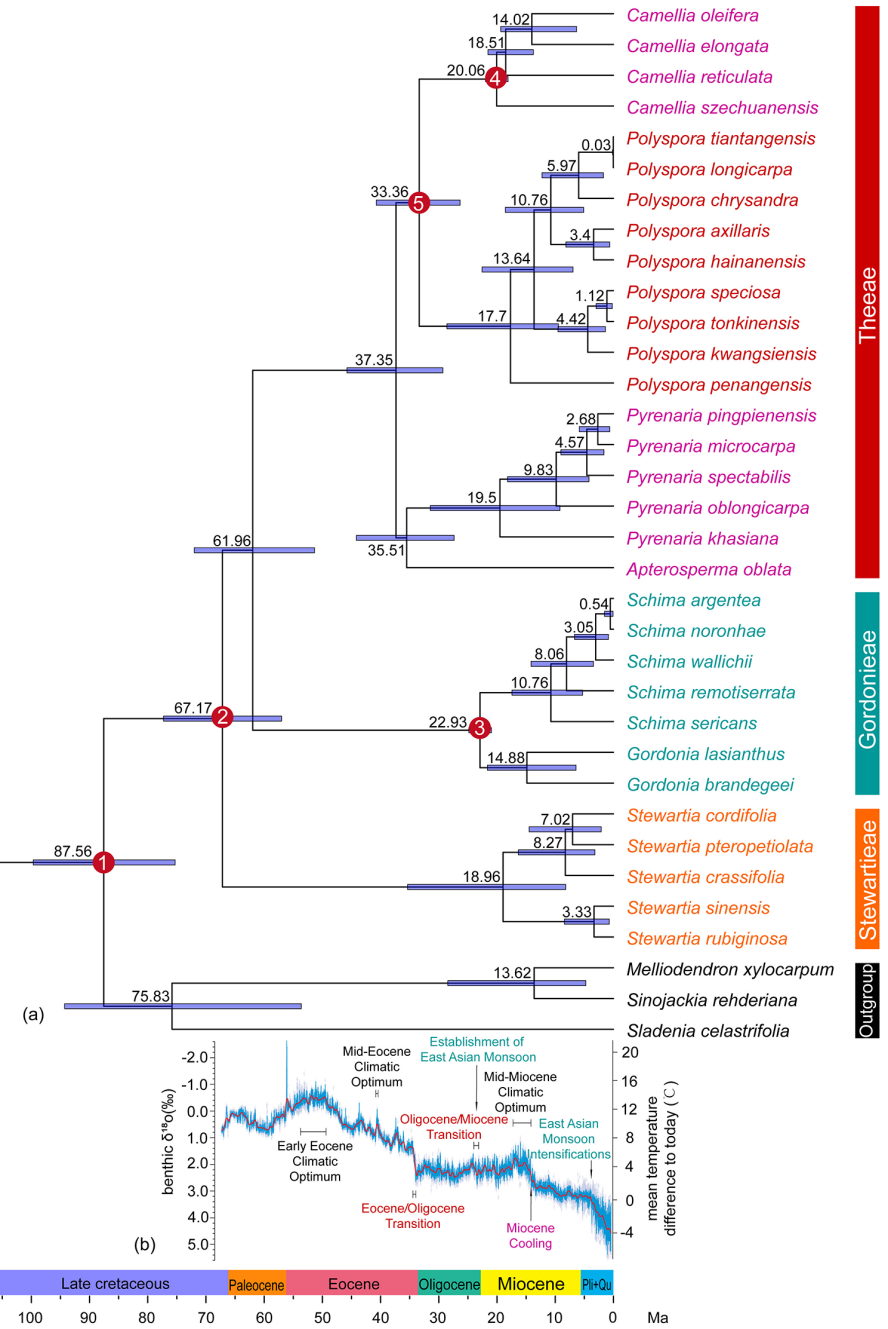
Chronological tree of the divergence of Theaceae species with five basic calibrations and a maximum restricted age of 109 Ma; **(a)** Chronological tree of species differentiation of Theaceae; **(b)** Global ocean surface temperature trends derived from benthic foraminiferal δ^18^O curves [8]. The numbers of the red nodes represent five fossil calibration points, as detailed in Table S1

We combined the chloroplast genome and ribosomal 18-26 S rRNA datasets because they are not significantly incongruent (Icong index was 1.78, *P* < 0.05). Phylogenetic tree of the combined dataset revealed that the divergence relationships among species within the genus *Polyspora* were generally consistent with the chloroplast genome phylogenetic tree. However, the divergence relationships and branch lengths of other taxa within Theaceae differed from those using the two datasets alone (Fig. 3, Figure S3). Consequently, the combined dataset provided more accurate phylogenetic relationships. On this basis, two maximum age limits were utilized for BEAST molecular clock estimation to generate two phylogenetic trees with divergence time calibration.

Under the maximum age limit of 109 Ma, the stem age of *Polyspora* was estimated to be 33.36 Ma, with a 95% highest posterior density (HPD) ranging from 26.34 to 46.75 Ma, and the crown age of *Polyspora* was 17.7 Ma (95% HPD: 9.46–28.61 Ma). Chinese *Polyspora* diverged in the middle Miocene at 13.64 Ma (95% HPD: 6.95–22.57 Ma) (Fig. 3).

Alternatively, under the maximum age limit of 125 Ma, the estimated stem age of *Polyspora* was 34.83 Ma (95% HPD: 27.31–42.30 Ma), the crown age of *Polyspora* was estimated to be 18.26 Ma (95% HPD: 11.05–28.38 Ma). Chinese *Polyspora* diverged in the middle Miocene at 14.48 Ma (95% HPD: 8.92–22.94 Ma) (Figure S4). The estimated times under the two maximum node age limits were close to each other, suggesting that increasing the node age limit had limited impact on the origin and divergence time of genus *Polyspora*.

### Diversity, genetic structure, and phylogeographical analysis of chloroplast combined sequences

The sequences of the three chloroplast genes were combined in the order of their placement in the chloroplast genome, *trn*H-*psb*A→*rpo*B-*trn*C→*pet*N-*psb*M. Chloroplast haplotype codes were denoted by “C”, and numbers were listed in the order of their detection in the population.

The total length of the combined sequence was 2,898 bp, of which *trn*H-*psb*A was 477 bp long, *rpo*B-*trn*C was 1,278 bp long, and *pet*N-*psb*M was 1,143 bp long. A total of 90 polymorphic sites were identified, consisting of 29 singleton variable sites and 61 parsimony informative sites. The nucleotide diversity (π) was calculated to be 0.01054. A total of 52 haplotypes were obtained, and the overall haplotype diversity (Hd) was 0.927 (Table S2). *Polyspora axillaris* and *P. speciosa* shared haplotype C11, while the other haplotypes were unique to each species. At the species level, the highest haplotype diversity and nucleotide diversity were found in *P. axillaris*, with Hd = 0.7746 and π = 0.00869. At the population level, the highest haplotype diversity and nucleotide diversity were found in the YP population of *P. longicarpa*, with Hd = 0.8333 and π = 0.00263 (Table S2).

AMOVA showed that 77.91% of genetic variation occurred interspecies, indicating a significant difference between species of *Polyspora*. Inter-population genetic variation accounted for 16.11% of the total, while intra-population genetic variation accounted for 5.98% (Table S3). Interpopulations gene flow in the genus *Polyspora* was calculated as Nm = 0.96. Permut software results showed that the average genetic diversity *hS* within the population was 0.398, and the total genetic diversity *hT* was 0.953. The total genetic diversity was higher than the inter-population genetic diversity, suggesting limited gene exchange between populations. The genetic differentiation coefficients *N*_ST_ and *G*_ST_ were calculated as 0.921 and 0.583, respectively, with *N*_ST_ significantly higher than *G*_ST_, indicating that genus *Polyspora* had a distinct phylogeographical structure.

At the species level, genetic variation in *P. axillaris* primarily occurred among populations (91.52%), with only 8.48% within populations. *Polyspora speciosa* also exhibited greater genetic variation among populations (70.8%), while 29.2% was observed within populations. However, the main genetic variations of *P. chrysandra*, *P. longicarpa*, and *P. hainanensis* occurred within populations (Table S3). The phylogeographical structure of *P. axillaris* (*N*_ST_=0.933, *G*_ST_=0.755, *P* < 0.05) and *P. speciosa* (*N*_ST_ =0.717, *G*_ST_ =0.454, *P* < 0.05) were evident. *Polyspora chrysandra* (*N*_ST_ =0.281, *G*_ST_ =0.434), with *N*_ST_< *G*_ST_, lacked a phylogeographic structure.

**Fig. 4:**
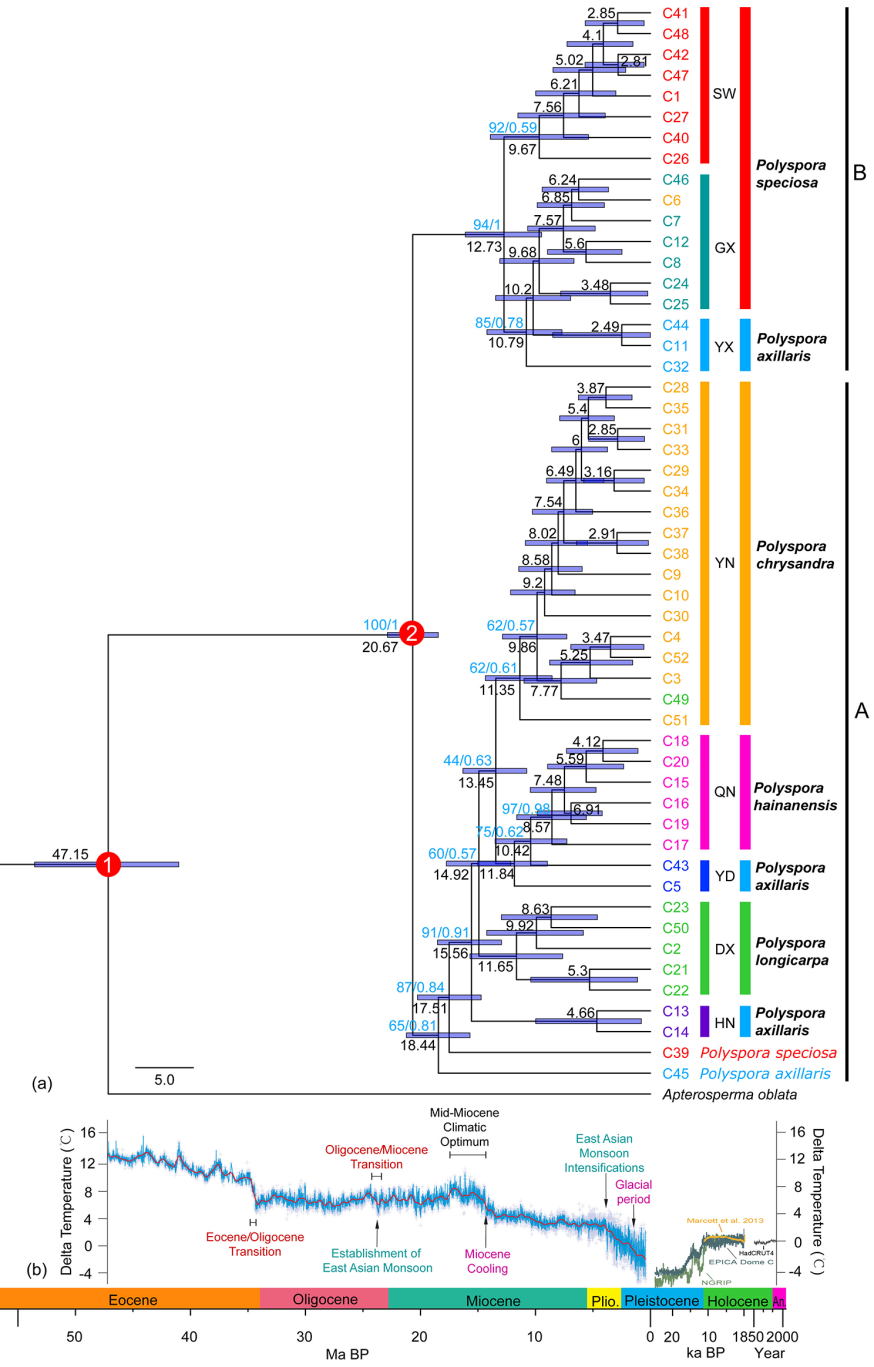
Phylogenetic tree of *Polyspora* based on combined chloroplast regions (*trn*H-*psb*A, *rpo*B-*trn*C, and *pet*N-*psb*M); **(a)** Haplotype phylogenetic tree; **(b)** Past global mean temperature trends [8]. The light blue progress bar on each node is 95% HPD confidence range, the black number below branches are the median differentiation time, the blue number above branches are the support rate of ML tree and the posterior probability of BI tree, and the red circle node is the secondary calibration node

**Fig. 5:**
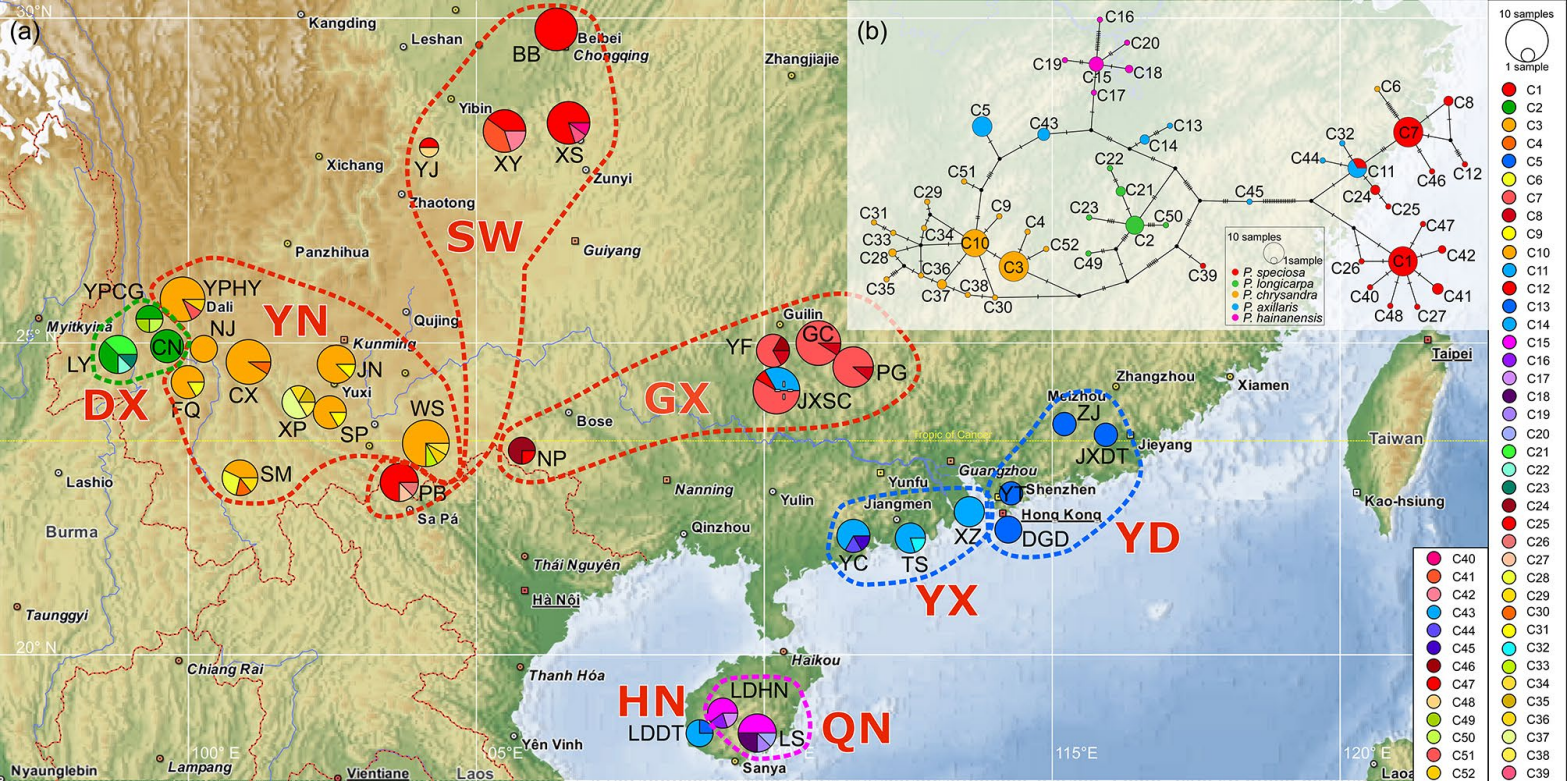
Haplotype geographical distribution and interspecies network of combined cpDNA (*trn*H-*psb*A, *rpo*B-*trn*C, and *pet*N-*psb*M) in 32 populations of *Polyspora*. **(a)** Haplotype map; **(b)** Interspecies haplotype network. Size of the circle is proportional to the relative frequency of the haplotype. The map was drawn by the authors using the popart-1.7 software (http://popart.otago.ac.nz/), and the base map was the software’s built-in map

Using *Apterosperma oblata* as the outgroup, 52 haplotypes were constructed by both ML and BI methods. The phylogenetic trees obtained from the two methods were basically consistent on the main clades, secondary and tertiary branches, and main branchlets. The phylogenetic tree initially bifurcated the *Polyspora* haplotypes into clades A and B, with a support rate of 100% between the two clades (Fig. 4). Clade A comprised predominantly four species: *P. chrysandra*, *P. hainanensis*, *P. axillaris*, and *P. longicarpa*. Clade B consisted mainly of *P. speciosa* and *P. axillaris*. *Polyspora axillaris* was found in both clades, while other species exhibited only a few haplotypes nested in different lineages. Clade A first separated two ancient haplotypes C45 and C39, belonging to YC population of *P. axillaris* and XS population of *P. speciosa*. Subsequently, the divergences were as follows: Hainan lineage of *P. axillaris* (labeled as HN), *P. longicarpa* lineage (labeled as DX), *P. hainanensis* lineage (labeled as QN), and east Guangdong lineage of *P. axillaris* (labeled as YD). The final differentiation was Yunan lineage composed of 16 haplotypes of *P. chrysandra* and 1 haplotype of *P. longicarpa* (labeled YN). Clade B was also divided into two clades, with one tertiary clade further splitting into SW, GX and YX. SW consisted of 8 haplotypes of *P. speciosa* from the southwest lineage (populations located in Yunnan, Guizhou, Sichuan, and Chongqing). GX included 6 haplotypes of *P. speciosa* from the Guangxi lineage (all populations located in Guangxi), and one haplotype C6 of *P. chrysandra* from FQ population. YX included three haplotypes of *P. axillaris* from the western Guangdong lineage (all populations located in western Guangdong) (Figs. 4 and 5a). Specifically, *P. axillaris* exhibited three lineages: YX, YD, and HN, while *P. speciosa* showed two lineages: SW and GX. *Polyspora chrysandra*, *P. longicarpa*, and *P. hainanensis* did not display any discernible geographical structure.

The haplotype network diagram was generally consistent with the haplotypes phylogenetic tree. The diagram was centered around the haplotype C45 and divided into two major clades. Among the minor clades, C1, C2, C3, C5, C7, C10, C11, and C15 had a higher frequency of distribution, most of which were located at the center of their respective clades (Fig. 5b, Figure S5). These haplotypes were speculated to be the ancient haplotypes of *Polyspora* in China. The haplotype of *P. axillaris* had the widest distribution and occurred in both major clades. *Polyspora speciosa* was distributed in two clades, with haplotypes C1 and C7 as the centers forming two stellate structural clades. Haplotype C7 was formed by the shared haplotype C11 of *P. axillaris* and *P. speciosa* through three steps of differentiation. *Polyspora chrysandra* also included two stellate clades, centered around C3 and C10, respectively. *Polyspora longicarpa* was a stellate structural clade composed of C2 as the center haplotype. *Polyspora hainanensis* was a stellate clade with C15 as the centered haplotype.

The phylogenetic differentiation time of Theaceae was used as a secondary calibration. *Polyspora* and *Apterosperma* both originated at 37.35 Ma and diverged at 13.64 Ma. The average molecular variation rate of Theaceae was 1.79E-4 s/s/y, and the divergence time of chloroplast haplotypes was estimated using the BEAST molecular clock. The results showed that the divergence time of clade A and B was approximately 20.67 Ma (95% HPD: 18.45–22.88 Ma), and the oldest haplotype C45 of Chinese *Polyspora* diverged at about 18.44 Ma (95% HPD: 15.69–21.22 Ma). The divergence time of *P. speciosa* was estimated to be about 12.73 Ma (95% HPD: 9.47–16.09 Ma), the divergence time of GX lineage was about 10.2 Ma (95% HPD: 6.94–13.47 Ma), and the divergence time of SW lineage was approximately 9.67 Ma (95% HPD: 5.39–13.92 Ma). The divergence time of YX lineage of *P. axillaris* was about 10.79 Ma (95% HPD: 7.69–14.22 Ma), the YD lineage was about 11.84 Ma (95% HPD: 8.96–15.03 Ma), and the HN lineage was the latest, estimated to be about 4.66 Ma (95% HPD: 0.8–9.98 Ma). *Polyspora chrysandra* diverged at 13.45 Ma (95% HPD: 10.76–16.31 Ma). *Polyspora longicarpa* diverged at 11.65 Ma (95% HPD: 7.64–15.7 Ma). *Polyspora hainanensis* diverged at 8.57 Ma (95% HPD: 5.57–11.63 Ma) (Fig. 4).

Mantel correlation test showed that the linear relationship between genetic distance and geographic distance of genus *Polyspora* was y = 0.00763 + 2.76 × 10^− 6^x, R^2^ = 0.02, *P* < 0.001. With the increase of geographical distance, genetic distance increased correspondingly, showed a highly significant positive correlation (Figure S6). Similarly, *P. speciosa* demonstrated a highly significant positive correlation between genetic distance and geographical distance, y = − 0.000252 + 3.37 × 10^− 6^x, R^2^ = 0.54, *P* < 0.001. On the other hand, there were no strong correlation observed between genetic distance and geographical distance in *P. axillaris* and *P. chrysandra* (Figure S6).

To investigate the population dynamic history of the genus *Polyspora* and species within the genus, neutral testing and mismatch analysis were performed using chloroplast associated sequences. Neutral test data of *Polyspora* showed that Tajima’s D = 0.82434 (*P* > 0.10), Fu and Li’s D=-2.91364 (*P* < 0.05), and Fu and Li’s F=-1.39655 (*P* > 0.10), indicating that neutral evolution was not rejected. In the mismatch analysis, the observation curves of genus *Polyspora*, *P. axillaris*, and *P. speciosa* showed distinct multiple peaks, indicating that these taxa may not had undergone significant expansion recently (Figure S7). Conversely, *P. chrysandra* exhibited a unimodal curve, with the neutral test result Tajima’s D=-2.50609 (*P* < 0.001) was extremely significant. Additionally, Fu and Li’s D=-5.73205 (*P* < 0.05), Fu and Li’s F=-5.37156 (*P* < 0.05), both deemed statistically significant. Collectively, these findings indicated that *P. chrysandra* had experienced recent population expansion. By integrating the corresponding Tau values and BEAST molecular clock results, the population expansion time was estimated to be about 3.26 Ma, predating the last glacial maximum. *Polyspora longicarpa* and *P. hainanensis* also exhibited unimodal curves, but the available sample size of these two species were small, and more samples and data were needed for analysis and research.

## Discussion

### Phylogenetic and evolutionary history of genus ***Polyspora***

*Polyspora axillaris* and *P. hainanensis* are sisters, which is consistent with their geographical distribution. *Polyspora axillaris* is mainly distributed in Guangdong, Taiwan, Hong Kong and Hainan, while *P. hainanensis* is exclusively found on Hainan Island. Three species of *P. speciosa*, *P. kwangsiensis*, and *P. tonkinensis* formed a monophyletic lineage, which are homologous and have minimal morphological distinctions. We propose merging these species into a single entity based on this resemblance. Due to the earliest publication of *P. speciosa* (1900) [7], the merged species ought to be named *P. speciosa*. *Polyspora chrysandra* and *P. dalgleishiana* formed sister clades, which are also two geographically similar species. *Polyspora chrysandra* predominantly inhabits Yunnan Province, whereas *P. dalgleishiana* can be found in Vietnam, bordering China’s Yunnan Province. *Polyspora tiantangensis* was found to be closely associated with *P. longicarpa* due to their shared geographical distribution and similar morphological characteristics. Based on their comprehensive morphological and molecular results, they should be treated as the same species. On the other hand, *Polyspora penangensis* from Malaysia formed a distinct clade. Currently, 47 species of *Polyspora* have been accepted. However, the species coverage and molecular data employed in this study were limited. Although these findings could shed light on the interspecies relationships among Chinese *Polyspora* species, they may not elucidate the phylogenetic relationships of the whole genus. To address this matter, future research should incorporate as many *Polyspora* species as possible.

Our species divergence times indicate that Chinese *Polyspora* species diverged in the middle Miocene at 13.64 Ma. This time is earlier than the estimation by Yu et al. [9] and close to the estimation by Rose et al. [10]. Furthermore, the intra-genus taxa divergence time is in line with the estimation by Ryu et al. [11].

*Polyspora* is predominantly found in the tropical regions of South and Southeast Asia. This study only focused species native to China, with relatively few species sampled within the genus and fewer nuclear genes selected. Therefore, the possible origin site and migration route of this genus were not investigated, and the divergence history of this genus could not be accurately depicted. Currently, fossil records of *Polyspora* have been discovered in Europe, suggesting that China may not be the origin of *Polyspora*, as indicated by the phylogenetic and divergence time series maps. Although no fossils have been found from the modern distribution area of genus *Polyspora*, it can be inferred that *Polyspora* had a broader historical distribution based on fossils identified in the Eocene, Oligocene, and Miocene strata of Europe. Plate tectonic theory and plant fossils from the early Eocene of India suggest that the tropical angiosperms of southern Asian originated from the Deccan Plate (present-day India and Sri Lanka). Approximately 45 million years ago, after the collision of the Deccan Plate with Eurasian continent, plants from the Deccan Plate migrated to Eurasia during the Eocene, Oligocene and Miocene periods [12, 13]. Some evidence suggests that the Indo-Malaysian flora was widespread during this plate collision, extending all the way to Europe and Greenland [14]. The genus *Polyspora* may have originated and spread to Europe after the collision of the two tectonic plates. As the climate cooled, the habitat of *Polyspora*, which used to be widely distributed in Eurasia, gradually diminished until eventually disappearing. The warm and humid regions of South and Southeast Asia became the modern distribution centers of this genus. Hence, it is essential to conduct phylogenetic analyses of the entire genus, utilizing more comprehensive genomic methodologies in conjunction with entire species distributions. Special attention should be given to Vietnam, where relict species of the genus may still exist, as well as Malaysia, Indonesia, and other countries with high species richness of the genus. This approach will provide a deeper understanding of the speciation and divergence mechanism of *Polyspora*.

### Reasons for the formation of phylogeographic patterns of genus ***Polyspora*** in China

The chloroplast haplotype network and phylogenetic tree revealed the presence of two principal clades and eight distinct lineages within the genus *Polyspora* in China. The estimated divergence time for the two major clades was 20.67 Ma (95% HPD: 18.45–22.88 Ma), which fell within the 95% HPD range (6.95–22.57 Ma) of the divergence time of Chinese *Polyspora*, suggesting an divergence in the early Miocene which aligns with the initial divergence time of the genus *Polyspora*.

The late Oligocene to early Miocene was the termination of the India-Eurasia collision and plate subduction, resulting in intensified orogeny. Among the regions affected, China experienced the most significant impact from the Himalayan orogeny [15]. Approximately 20 Ma ago, the Himalayas underwent rapid uplift, soaring from an elevation of less than 3,000 m to near their current altitude [16]. This abrupt elevation change exerted a profound influence on the atmospheric circulation in Asia, prompting a shift from the original planetary wind system to a monsoon wind regime within Chinese Mainland. The paleoclimate change reached its peak and gradually transitioned towards the current climate [17]. As a direct consequence of the Himalayan uplift, China began to exhibit a high-altitude climate in the southwest, a temperate arid climate in the northwest, and a warm-temperate, subtropical, and tropical humid climate in the eastern regions from north to south [18]. Climate pattern alterations gave rise to interspecific differentiation, fostering the development of species diversity within genus *Polyspora*. Notably, two fossil records, *Polyspora hradekensis* [19] and *Polyspora europaea* [20], originate from this period. The fossils of *P. hradekensis* bear a striking resemblance to the extant *P. balansae* species, which is a likely relict species from Vietnam. Considering the geographical proximity between Vietnam and China, together with the similarities in the initial divergence of Chinese *Polyspora*’s cpDNA haplotypes and the whole genus, we hypothesize that the Sino-Vietnam border may represent the divergence center of Chinese *Polyspora*.

Clade A exhibited the earliest divergence, resulting in the emergence of haplotype C45 of *P. axillaris* at 18.44 Ma (95%HPD: 15.69–21.22 Ma), and haplotype C39 of *P. speciosa* at 17.51 Ma (95%HPD: 14.72–20.25 Ma). Subsequently, the divergence time was essentially synchronized with clade B around 12–15 Ma, these two clades gradually diversified into eight lineages. Among these lineages, the divergence between the two lineages of *P. speciosa* occurred during the middle Miocene at 12.73 Ma (95%HPD: 9.47–16.09 Ma). However, the three lineages of *P. axillaris* formed at different intervals, with the YD and YX lineages diverging during the middle and late Miocene, while the HN lineage diverged during the Pliocene. Additionally, *P. chrydandra*, *P. longicarpa*, and *P. hainanensis* also diverged during the middle and late Miocene. The available fossil record of the genus *Polyspora* includes five specimens from the Miocene, of which four are from the late Miocene. This corroborates the estimated time of divergence of cpDNA haplotype lineages determined by the BEAST molecular clock. Thus, we suggest that the species lineages and major geographic lineages of the genus *Polyspora* in China were formed prior to the Quaternary glacial period. During the mid-Miocene, there was a temporary interruption of the long-term cooling trend of the Cenozoic, resulting in a short-term climatic optimum known as the Mid-Miocene Climatic Optimum (MMCO, 15–17 Ma) [21]. This climate phase provided favorable conditions for the genetic divergence of *Polyspora*. The cpDNA haplotypes of *Polyspora* in China primarily diverged during the late Miocene, around 6 million years before present. Following the MMCO period, the climate continued to cool. By the late Miocene in China, an eastern monsoon-northwestern arid climatic pattern had been established, and there were already clear temperate-subtropical-tropical climatic distinctions from north to south. As a response to these changes in the climate pattern, the species of *Polyspora* experienced intraspecific differentiation, leading to the gradual emergence of genetic diversity.

### Glacial refugia of ***Polyspora*** in China

The gene flow resulting from seed dispersal is lower than that caused by pollen dispersal. Maternally inherited cpDNA molecular markers can not only reflect the phylogeographical pattern of species, but also reproduce the glacial refugia of plants and their post-glacial migration routes [22, 23]. Populations situated in glacial refugia usually exhibit high levels of genetic diversity, haplotype polymorphism, and more endemic haplotypes [24, 25]. Furthermore, these areas are often equipped with geo-ecological conditions that enable them to withstand unfavourable environmental factors [26]. In summary, the genus *Polyspora* may have multiple refugia in China, and these may vary according to species (Figure S8). Based on the haplotype distribution characteristics observed in this study and the findings from our team’s previous species distribution model [4], the inferred glacial refugia for each species are as follows: *Polyspora speciosa* was found to have refuged at the northeast edge of the Yunnan-Guizhou Plateau and Nanling Mountain; *P. chrysandra* at the Wuliang and Ailao Mountains; *P. longicarpa* at the Gaoligong Mountain; and *P. axillaris* at the Lianhua Mountain in eastern Guangdong (Figure S8).

The Pleistocene Ice Age had a profound impact on the spatial distribution pattern and genetic structure of modern organisms [27]. Numerous plant taxa distributed in subtropical EBLFs sought refuge in situ in multiple refugia, such as the Hengduan Mountains, Yunnan-Guizhou Plateau, Qinba Mountains, and Nanling Mountains [28]. Most cpDNA haplotypes of Chinese *Polyspora* had already formed prior to the last glacial maximum, and the characteristics of glacial refugia align with the prevalent in situ survival pattern. The main refugia of each species are also similar to relevant research results. Simultaneously, *Polyspora* also has multiple refugia within inland China, as populations stemming from the same refugium show more homogeneous haplotype compositions [22, 29]. *Polyspora* displays a wide array of haplotypes, with minimal cpDNA haplotype sharing between species and regions. The existence of multiple refugia and low interspecific gene flow may be one of the reasons for the current geographical distribution pattern and relatively independent interspecific distribution.

Neutrality test showed that the overall level of *Polyspora* was consistent with neutral evolution. Additionally, the mismatch analysis displayed a clear multimodal curve for *Polyspora*, indicating that it had not experienced recent expansion. According to the divergence time estimated by BEAST, the main lineage of *Polyspora* diverged during the last interglacial period (13 − 7.5 Ma), with most haplotypes diverging earlier than the last glacial maximum. The small-range expansion events of each species indicated by the starburst-radiating haplotype network were basically remote expansions. During the Quaternary glacial period, geological and climatic turbulence led to the re-concentration of haplotype diversity and the subsequent formation of glacial refugia.

The glacial period had minimal influence on the distribution pattern of the entire genus. At the species level, although the neutral test and mismatch analysis of *P. chrysandra* indicated recent expansion, the expansion occurred before the last glacial maximum. The results at the overall level, species level and lineage level of Chinese *Polyspora* collectively demonstrate that the primary geographical distribution pattern of the genus had already established prior to the Ice Age. The historical dynamics of the genus have been relatively stable and maintain stability during periods of climate turbulence.

Among these refugia, the border among Yunnan, Guangxi, and Vietnam was inferred by the ecological niche model to be the species divergence center of Chinese *Polyspora* [4]. During the glacial period, four *Polyspora* species had different scales of distributions in this region, suggesting it may also serve as the genetic mixing center of genus *Polyspora* (Figure S8). This region belongs to the transition zone between subtropical and tropical, mainly characterized by karst landforms and comprises many relatively independent microhabitats. The dominant vegetation types consist of northern tropical monsoon rainforests, which experienced minimal climate turbulence during the Pleistocene glacial period. The unique geographical location and ecological environment, as well as stable and humid climate, provided ideal conditions for tropical and subtropical species to seek refuge and exchange genes during the glacial period. The Nanling Mountain Range is speculated to be the refuge of GX lineage of *P. speciosa* based on molecular phylogeography, while Lianhua Mountain Range is considered to be the refuge of *P. axillaris* according to both methods. These two mountains are close to each other and serve as transition zone between subtropics and tropics. Phylogeography studies have demonstrated that the Nanling Corridor played a crucial role as a stepping stone and dispersal channel for species during the late Quaternary, thereby facilitating the exchange of genetic resources between the eastern Yunnan-Guizhou Plateau and the eastern subtropical mountains [30–34]. The glacial refugia of SW lineage of *P. speciosa* are located in the eastern Yunnan-Guizhou Plateau. On the other hand, the glacial refugia of GX lineage are located in the western mountains of Nanling range. The nucleo-plastid conflict observed in the phylogeny of *Polyspora* primarily stems from the unclear evolutionary relationship between *P. speciosa* and *P. axillaris* (Fig. 2). According to the phylogenetic tree established by Zhang (2022) using 610 low-copy nuclear genes, *P. speciosa* and *P. axillaris* cluster into a monophyletic lineage [35], which share a recent common ancestor. Phylogeography studies utilizing chloroplast haplotypes have further revealed that the two species share the haplotype C11, which is the only interspecific shared cpDNA haplotype in Chinese *Polyspora*. This haplotype is present in the GX lineage of *P. speciosa* and the YX lineage of *P. axillaris*, both of which are situated in the vicinity of Nanling Mountains. Nanling Corridor was frequently inhabited by coniferous forests or EBLFs during the Quaternary climate turbulence [36]. The phylogeographic relationship between *P. speciosa* and *P. axillaris* suggests that the two species may have fixed genetic polymorphisms from a common ancestor and maintained secondary contact during the glacial periods after their speciation. The western Nanling Corridor serves as a genetic mixing center for the two species, which may be attributed to incomplete lineage sorting or gene introgression caused by secondary contact since glacial period.

## Conclusion

The genus *Polyspora* was closely related to *Camellia*. The earliest lineages of *Polyspora* diverged in the low-latitude tropics, with Chinese *Polyspora* species diverging during the middle Miocene. The main geographical distribution pattern of Chinese *Polyspora* had already been established prior to the last glacial maximum, and the historical dynamics of the population remained relatively stable. The geological and climatic turbulence during the Quaternary glacial period had minimal influence on the distribution pattern of the genus. *Polyspora* exhibited multiple refugia during the last glacial maximum, and the genus adapted to the Quaternary climate turbulence by surviving in situ during glaciation. The Sino-Vietnam border and the Nanling corridor were identified as potential genetic mixing centers for *Polyspora*. Nanling Mountains provide good geographical conditions for genetic resource exchange between plants in the eastern Yunnan-Guizhou Plateau and the eastern subtropical mountains.

## Materials and methods

### Sample collection, DNA extraction, and sequence annotation

During phylogenetic analysis, samples were obtained based on Yang Shixiong’s (2005) taxonomic treatment of Chinese *Polyspora* [5]. Specifically, samples were collected from 8 type localities where *Polyspora* specimens were found, or from populations in close proximity to their respective type specimens with distinct species identification characteristics (Table S4). All voucher specimens were morphologically identified by Zhifeng Fan from Kunming University of Science and Technology, and deposited in the Herbarium of Southwest Forestry University, the deposition numbers were shown in Table S4. The annual leaves of sample plants were collected and dried on silica gel for DNA extraction. Total genomic DNA was extracted using the BIOFIT Plant DNA Extraction Kit (BIOFIT, Chengdu, China). DNA concentration, integrity, and purity were detected by the Agilent 5400 Fragment Analyzer (Santa Clara, California, USA). Illumina NovaSeq 6000 platform from Novogene (Beijing, China) was utilized for genome skimming. Chloroplast genomes and ribosomal 18-26 S rRNA assembly were carried out using Getorganelle [37]. Sequences annotation were performed using Geneious R11 (https://www.geneious.com/), with the reference genome *P. axillaris* (NC_035645). The annotated sequences were submitted to GenBank with the accession numbers presented in Table S4.

According to the APG III/IV taxonomic system, Theaceae can be divided into three tribes and nine genera, of which three tribes and six genera are present in China. We downloaded the chloroplast DNA (cpDNA) and ribosomal 18-26 S rRNA data of representative species of the six genera from GenBank, downloaded two foreign *Polyspora* species (*P. penangensis* (Ridl.) Niissalo & L.M.Choo and *P. dalgleishiana* (Craib) Orel, Peter G.Wilson, Curry & Luu) gene data, and also downloaded gene data of 2 representative species of genus *Gordonia*, with *Melliodendron xylocarpum* Hand.-Mazz. *Sinojackia rehderiana* Hu, and *Sladenia celastrifolia* Kurz as outgroups. Together with the 8 Chinese *Polyspora* species sequenced in this study, making a total of 35 species (Table S5). With this dataset, we reconstructed the molecular phylogenetic tree of Theaceae.

During the phylogeographic and genetic structure analyses, we integrated the eight domestic *Polyspora* species into five species based on the results of the phylogenetic analysis. Experimental samples were from 32 populations, comprising 8 populations of *P. axillaris*, 9 populations of *P. chrysandra*, 10 populations of *P. speciosa*, 3 populations of *P. longicarpa*, and 2 populations of *P. hainanensis*. From each population, we selected 12 individuals, except for the YF population of *P. speciosa* where there were only 7 individuals available. The selected individuals had a minimum distance of 50 m between them. In total, we used 379 individuals as experimental samples for molecular phylogeography. The sample sources are listed in Table S2. For the analysis of chloroplast haplotype evolutionary tree, *Apterosperma oblata* (NC_035641) was used as the outgroup.

### Phylogenetic analysis and estimation of divergence time

To construct the phylogenetic relationship of genus *Polyspora* and check its phylogenetic position in Theaceae, the maximum likelihood (ML) and Bayesian inference (BI) methods were used based on the chloroplast genome dataset, ribosomal 18-26 S rRNA dataset, tandem chloroplast genome (with one IR region removed) and ribosomal 18-26 S rRNA dataset, respectively. Topological congruence between chloroplast genome and ribosome phylogenies was evaluated using the Icong index [38]. The optimal evolutionary models for each dataset was selected using ModelFinder [39], based on the AKAIKE information criterion. The optimal model for both the cpDNA dataset and cpDNA + 18-26 S rRNA combined dataset was GTR + I + G, while the optimal model for ribosomal 18-26 S rRNA was GTR + I + G4 + F. Phylogenetic trees were generated on the CIPRES platform [40], employing both maximum likelihood and Bayesian methods. The ML tree was iterated with RAxML v8.2.12 for 1,000 generations. The BI tree was iteratively run for 2,000,000 generations using Markov chain Monte Carlo method (MCMC) with 4 hot chains, sampling once every 100 generations, discarding the initial 25% aging samples, and merging the remaining trees to obtain the final Bayesian consistency tree. Finally, the phylogenetic tree was viewed and decorated using FigTree v1.4.4 (http://tree.bio.ed.ac.uk/).

To infer species origin and divergence time in Theaceae and genus *Polyspora*, we conducted molecular clock estimation using BEAST software. Our approach involved constructing two phylogenetic trees, each incorporating calibrated divergence times based on five fossil records and two maximum age limits. The phylogenetic trees were built upon a combination of the chloroplast genome (with one IR region removed) and the ribosomal 18-26 S rRNA. Fossil information was shown in Table S1.

Fossil ages were utilized to constrain minimum ages. If the tree’s root height was unconstrained, the root age was unrealistically aged. 109 Ma is the maximum limit for the secondary calibration of 95% HPD for the crown of Ericales [41], and 125 Ma is the earliest fossil age for tricolpate pollen dicotyledonous plants [42]. Previous studies have shown that root constraints of 109 Ma and 125 Ma have minimal impact on node age, producing similar outcomes [9]. Therefore, we set 109 Ma as the maximum constraint for the root height and compared it with the constraint at 125 Ma. Stratigraphic boundary ages were based on the International Chronostratigraphic Table (version 2021) published by the International Commission on Stratigraphy [43].

We employed the uncorrelated relaxed clock model for molecular clock analysis using BEAST v1.10.4 [44]. The parameter file (.xml) was generated using BEAUTi v1.10.4, and the main parameter were set as follows: Base substitution model was GTR; Evolution rate (Clock. rate) 1.52E-8; Tree prior was Birth-Death Model; Divergence time calibration based on the above fossil points; MCMC: Runs total algebra 500 million generations, log parameters every 10,000 times. Tracer v1.7.2 [45] was used to check if the parameters converge and ensure that the ESS values of all parameters were greater than 200. The tree was obtained with TreeAnnotator v2.6.6, with a Burnin percentage of 10%, Target tree type of MCC tree, Node heights of Median heights.

### Chloroplast DNA amplification and sequencing

To identify the optimal sequence fragments for genetic analysis of Chinese *Polyspora*, two individuals were selected from 32 populations during the primer screening phase, and eight highly variable chloroplast fragments (*trn*H-*psb*A, *rpo*B-*trn*C, *ycf*1, *rps*16, *pet*N-*psb*M, *ndh*F-*rp*l32, *ycf*4-*cem*A, *ndh*A) were amplified from the previous study [46]. Polymerase chain reaction (PCR) was performed in a 50 µl solution system. The solution consisted of 45 µl of gold medal Mix 1×TSE101, 2 µl of forward and reverse primers, 1 µl of plant DNA template. The reaction was performed on a PCR amplification instrument (LongGene A300, Hangzhou, China), with the following procedures: (1) initial denaturation at 98 ℃ for 2 min, (2) 35 cycles: denaturation at 98 ℃ for 10 s, annealing for 10 s, extension at 72 ℃ for 20 s, (3) extension at 72 ℃ for 5 min, and (4) final storage at 4 ℃. Primer information were shown in Table S6.

PCR reaction products were qualified by agarose gel electrophoresis detection, and after purification, they were sequenced by ABI 3730xl Genetic Analyzer (Thermo Scientific, Waltham, Massachusetts, USA). After screening and comparison, three cpDNA fragments (*trn*H-*psb*A, *rpo*B-*trn*C, and *pet*N-*psb*M) exhibiting superior polymorphism and higher amplification success rate, were selected for phylogeography study. Large-scale amplification and sequencing were performed on 379 individuals from 32 populations using the above three primers. Fragments larger than 600 bp were sequenced bidirectionally, for which *trn*H-*psb*A unidirectional sequencing, and the remaining three fragments bidirectional sequencing. The sequencing work was completed by Tsingke Biotech Company (Beijing, China), and the sequences have been deposited in GenBank (*trn*H-*psb*A: OR692860-OR693157, *rpo*B-*trn*C: OR692301-OR692557, *pet*N-*psb*M: OR692558-OR692859).

### Genetic diversity, haplotype networks, and geographic analysis of haplotype lineages

The raw data was subjected to peak chart checking, bidirectional sequence splicing, and sequence alignment using Geneious R11 (http://www.geneious.com), and exported in FASTA format. The three chloroplast fragments were analyzed jointly in accordance with their arrangement order in the chloroplast genome. Polymorphic loci, nucleotide polymorphism (π), haplotype number and type, haplotype polymorphism (Hd), gene flow and genetic differentiation were analyzed by DNAsp v6 [47], and the haplotype information files were derived. Permut CpSSR 2.0 software was used to calculate total genetic diversity *hT* and intra-population mean genetic diversity *hS* [48].

Permut CpSSR 2.0 was also used to detect the genealogical geographic signals of genus *Polyspora*, to calculate the overall genetic differentiation coefficient *G*_ST_, and the genetic differentiation coefficient *N*_ST_, which was jointly influenced by haplotype frequency and genetic distance between haplotypes, with 1,000 substitution tests. If *N*_ST_ is significantly greater than *G*_ST_ (*P* < 0.05), it indicates that there is an obvious genealogical geographic structure among populations. Haplotype frequency was calculated by Arlequin v3.5.2.2 [49]. Haplotype network diagrams and haplotype geographical distribution maps were drawn with popart-1.7 (http://popart.otago.ac.nz/), the TCS network method was chosen [50], and manually adjusted and embellished in Adobe Illustrator CS6 software.

### Population genetic differentiation and genetic structure

Analysis of molecular variance (AMOVA) was performed using Arlequin v3.5.2.2 [49] to calculate variance of variation between interspecies, intraspecies and interpopulations, as well as population genetic differentiation coefficient *F*_ST_. Mantel test [51] was used to examine the correlation between population genetic distance and geographic distance.

### Intraspecific phylogenetic analysis

Maximum likelihood (ML) and Bayesian (BI) methods were employed to construct haplotype phylogenetic trees of genus *Polyspora*, respectively, and the trees were processed in the PhyloSuite platform [52]. The optimal nucleotide substitution model was selected based on Akaike information criterion (AIC) method using Modelfider [39]. The parameters were as follows: ML tree 1,000 bootstraps, BI tree ran for 1 million generations, sampled every 100 generations, discarded the initial 25% sampled data, and use the remaining trees to construct a 50% consistent evolutionary tree. Tree visualization and editing was performed in Figtree (http://tree.bio.ed.ac.uk/), tree integration and visualization was performed with Adobe Illustrator CS6 software.

Chloroplast haplotype divergence time was estimated by BEAST v1.10.4 [44]. The parameters were as follows: uncorrelated relaxed clock model, random starting tree, substitution model was GTR + Gamma, tree prior was Yule Model, Markov chain length (MCMC) was 10 million generations, sampling every 1,000 generations. According to the results of phylogenetic analysis, two correction points were set, the root node (divergence time between *Polyspora* and *Apterosperma*) was 37.35 Ma, the divergence time of Chinese *Polyspora* was 13.64 Ma, and the clock rate was set to 1.79E-4. The method of parameters convergence checked and haplotype phylogenetic tree obtained were the same as *Phylogenetic Analysis and Estimation of Divergence Time*.

### Population dynamic history

Neutrality tests and mismatch analyses were performed using DNAsp v6 [47]. During the neutral tests, three values of Tajima’s D [53], Fu&Li’s Dand Fu&Li’s F [54] were calculated simultaneously. When Tajima’s D and Fu&Li’s D are significantly less than 0, it indicates that the population may have experience recent expansion or directional selection. Fu Li’s D&F test is more sensitive than Tajima’s D. In mismatch analyses, a unimodal distribution indicates recent population expansion, while bimodal or multimodal distributions indicate dynamic equilibrium or a declining trend in population. For lineages with significant expansion recently, the population expansion time was estimated based on their Tau value and evolutionary rate.

### Electronic supplementary material

Below is the link to the electronic supplementary material.

Supplementary Material 1

Supplementary Material 2

Supplementary Material 3

Supplementary Material 4

Supplementary Material 5

Supplementary Material 6

Supplementary Material 7

Supplementary Material 8

Supplementary Material 9

Supplementary Material 10

## Data Availability

The chloroplast genome sequences, ribosome 18-26 S nrRNA sequences, and the three cpDNA fragments sequences (trnH-psbA, rpoB-trnC, and petN-psbM) were submitted to GenBank (https://www.ncbi.nlm.nih.gov/). The accession numbers of cp. genome and ribosome 18-26 S nrRNA can be found in Table S3. The accession numbers of three cpDNA fragments are: trnH-psbA: OR692860-OR693157, rpoB-trnC: OR692301-OR692557, petN-psbM: OR692558-OR692859.
