# Supplementary figures and images for: Phylogeographical and population genetics of Polyspora sweet in China provides insights into its phylogenetic evolution and subtropical dispersal
Source: BMC Plant Biol. 2024 Feb 6;24:89. doi: 10.1186/s12870-024-04783-5 (PMC10845455; doi:10.1186/s12870-024-04783-5)

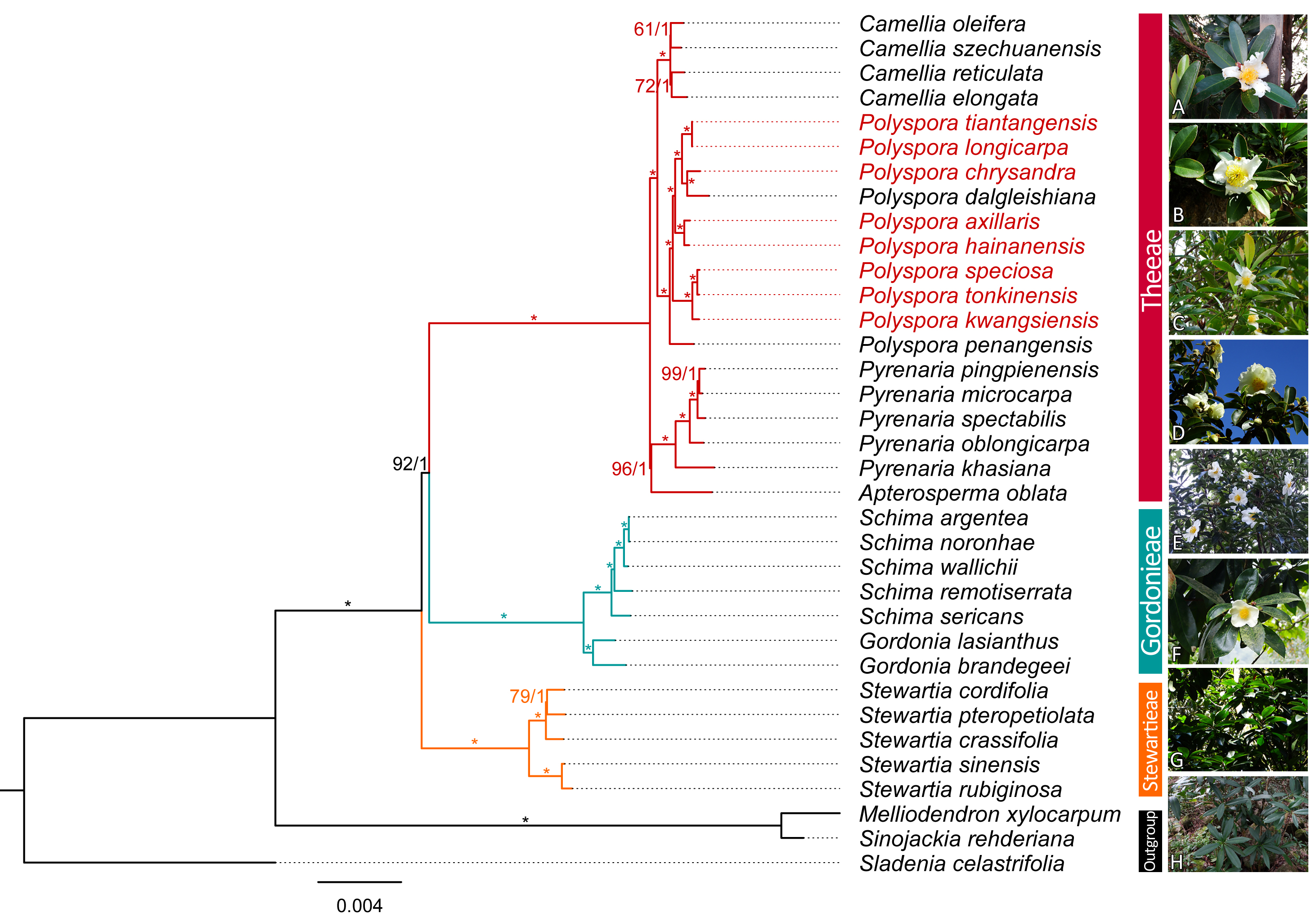

Supplement: Supplementary file 1 — Supplementary Material 1 [file 12870_2024_4783_MOESM1_ESM.jpg]

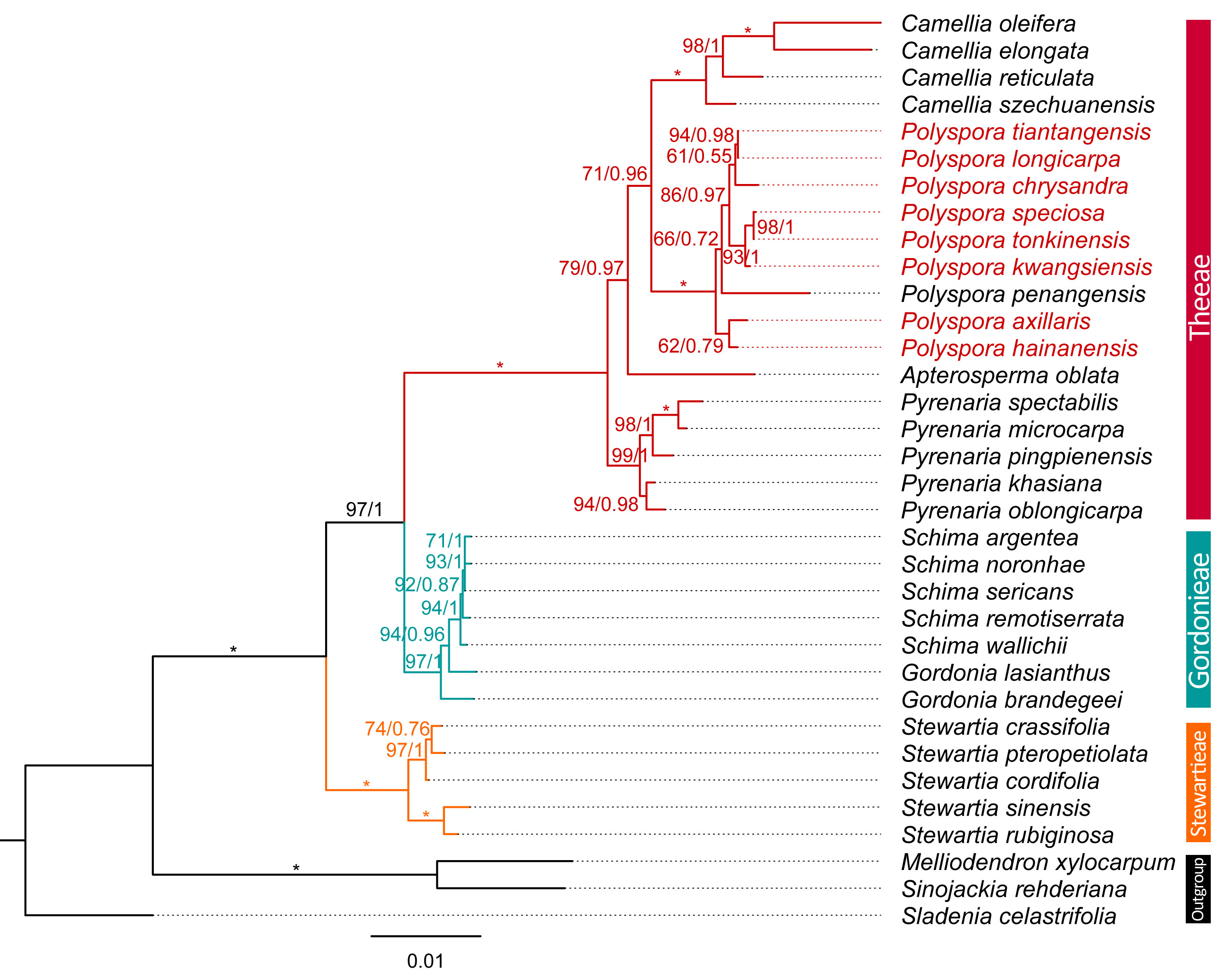

Supplement: Supplementary file 2 — Supplementary Material 2 [file 12870_2024_4783_MOESM2_ESM.jpg]

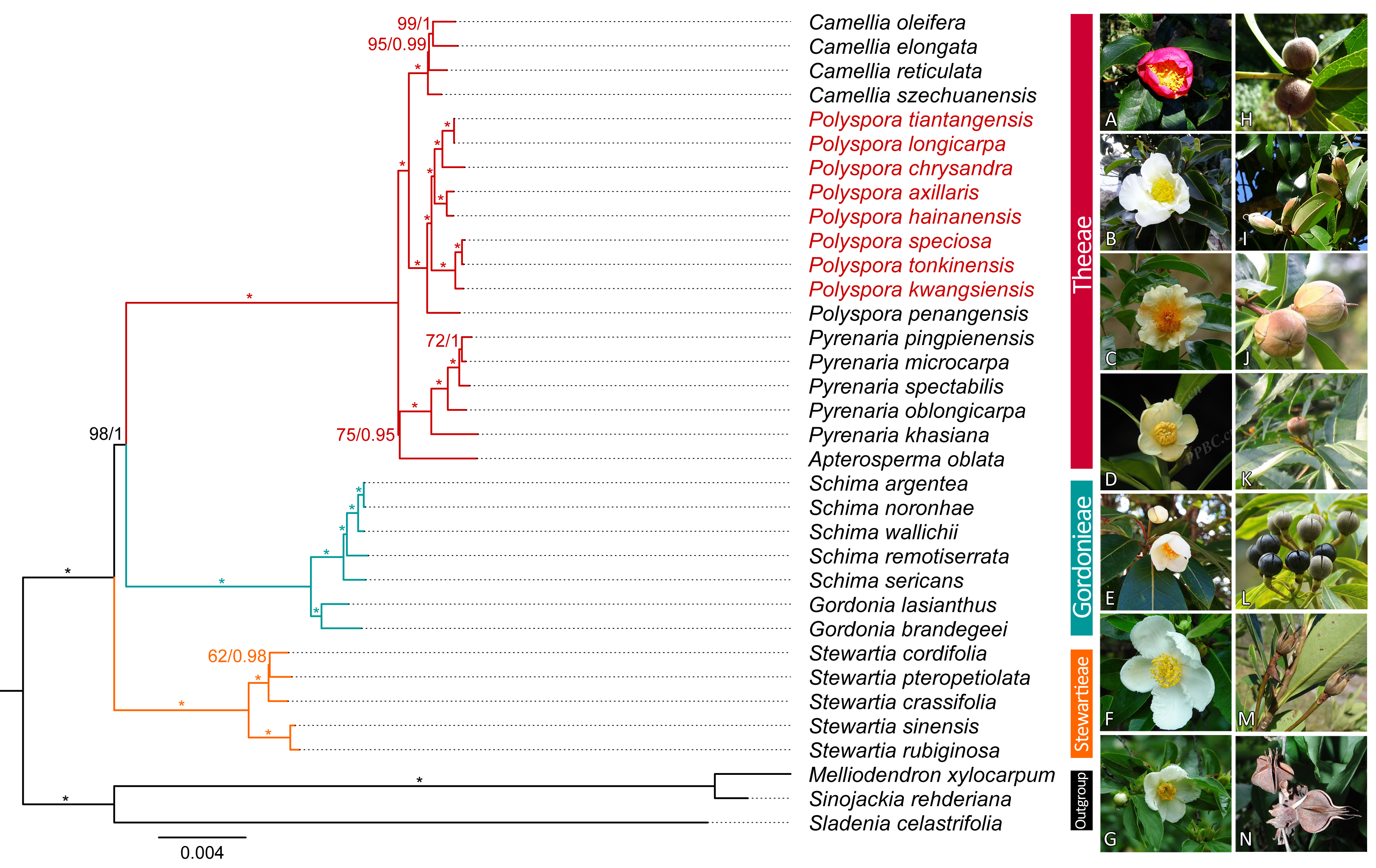

Supplement: Supplementary file 3 — Supplementary Material 3 [file 12870_2024_4783_MOESM3_ESM.jpg]

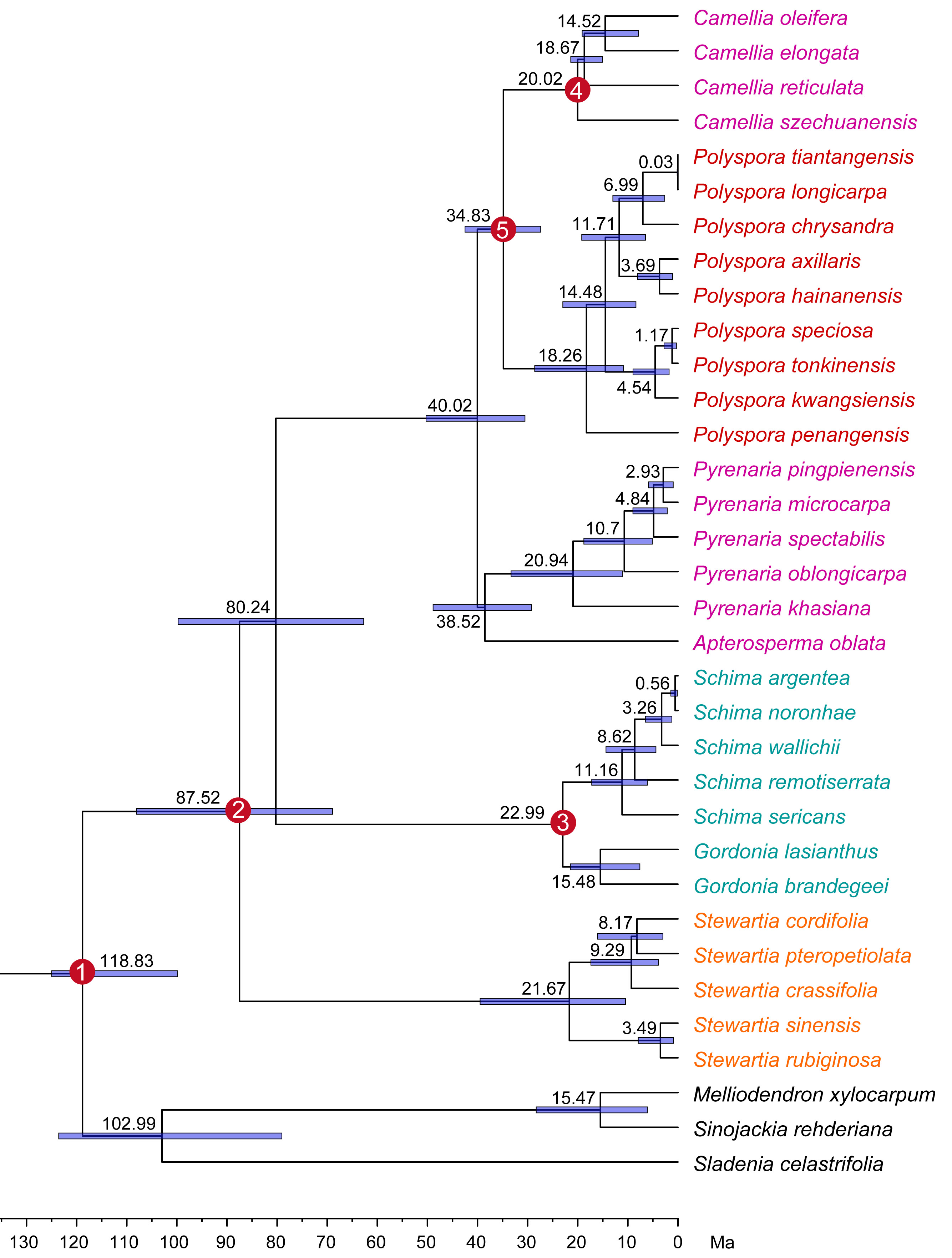

Supplement: Supplementary file 4 — Supplementary Material 4 [file 12870_2024_4783_MOESM4_ESM.jpg]

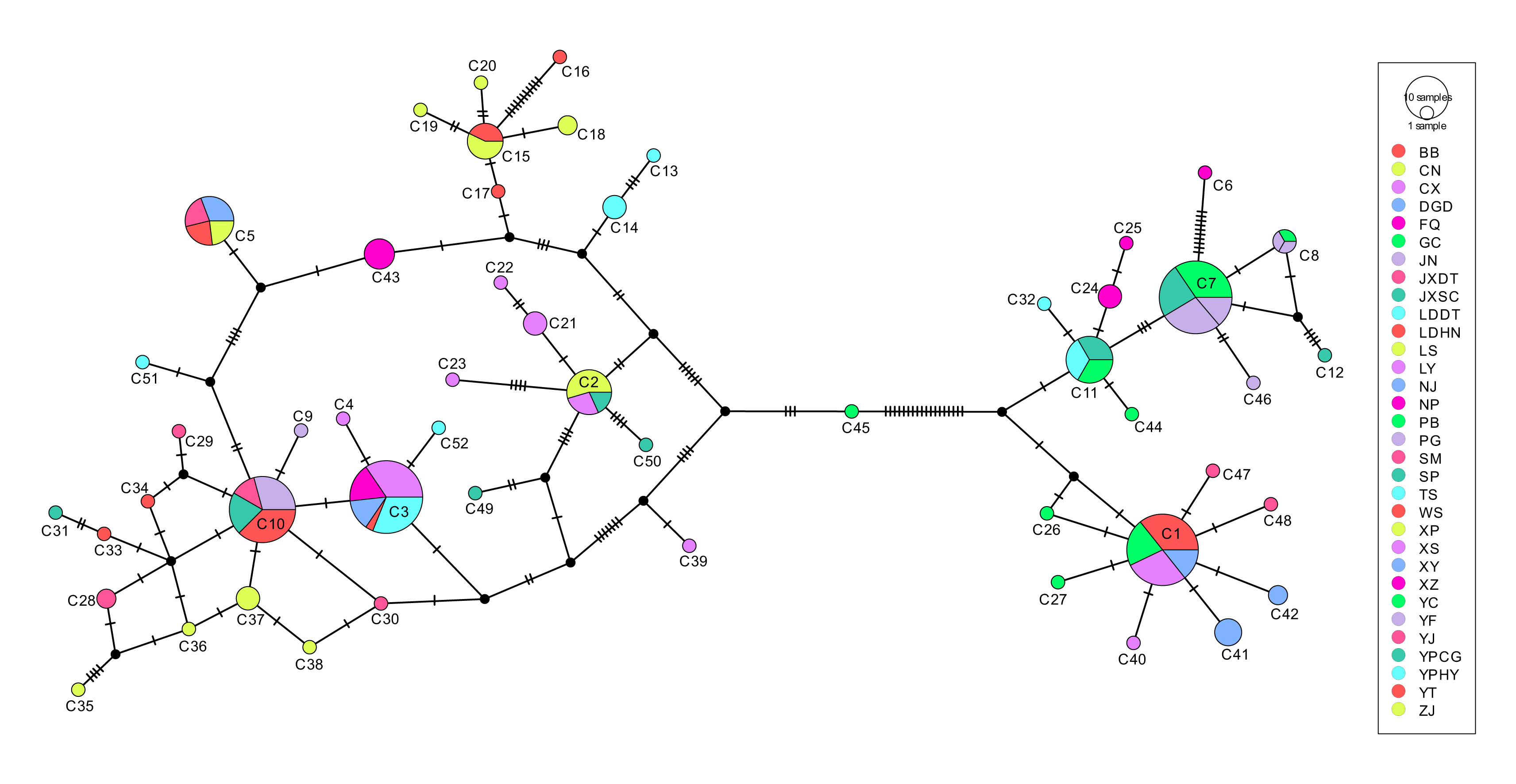

Supplement: Supplementary file 5 — Supplementary Material 5 [file 12870_2024_4783_MOESM5_ESM.jpg]

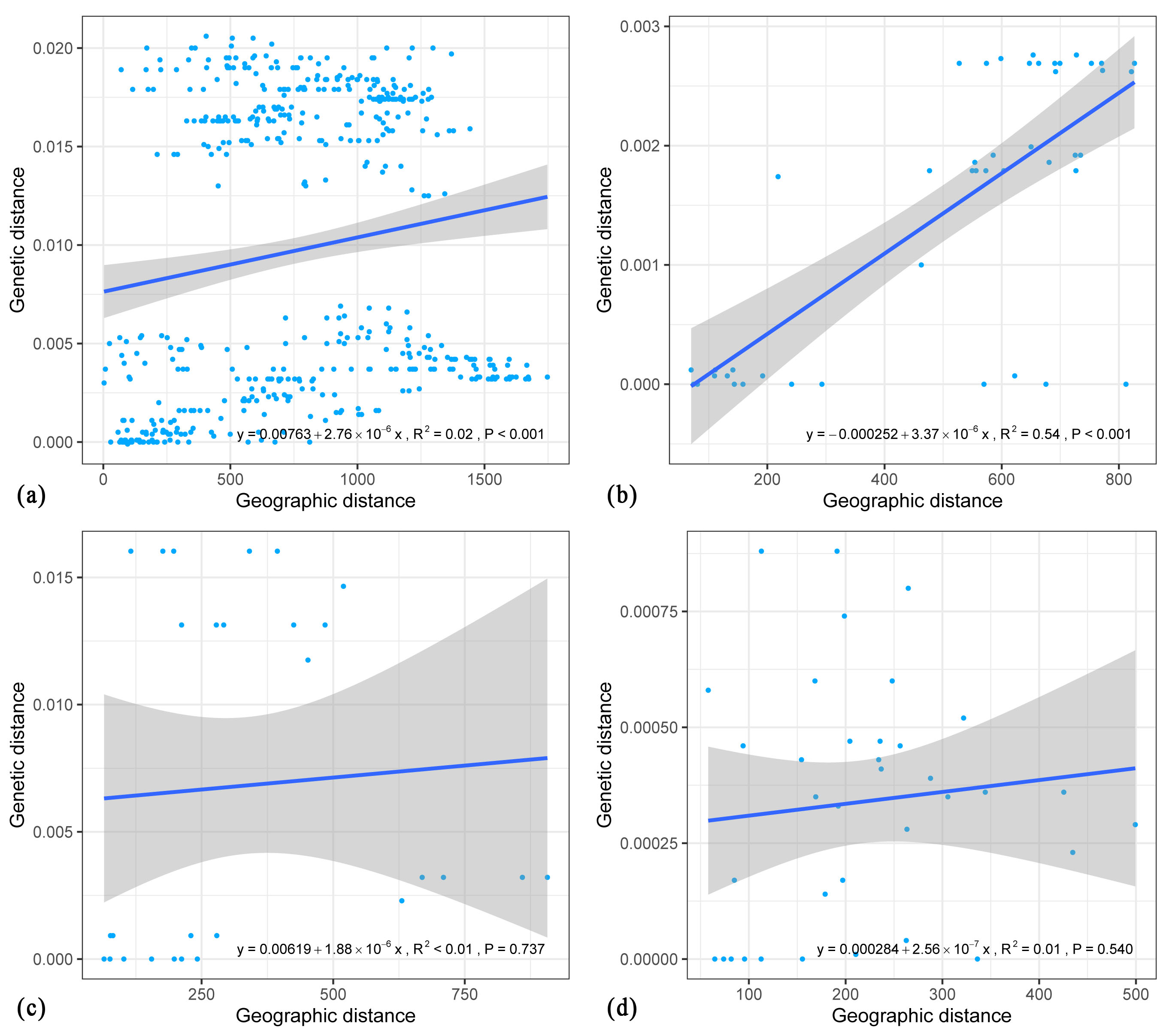

Supplement: Supplementary file 6 — Supplementary Material 6 [file 12870_2024_4783_MOESM6_ESM.jpg]

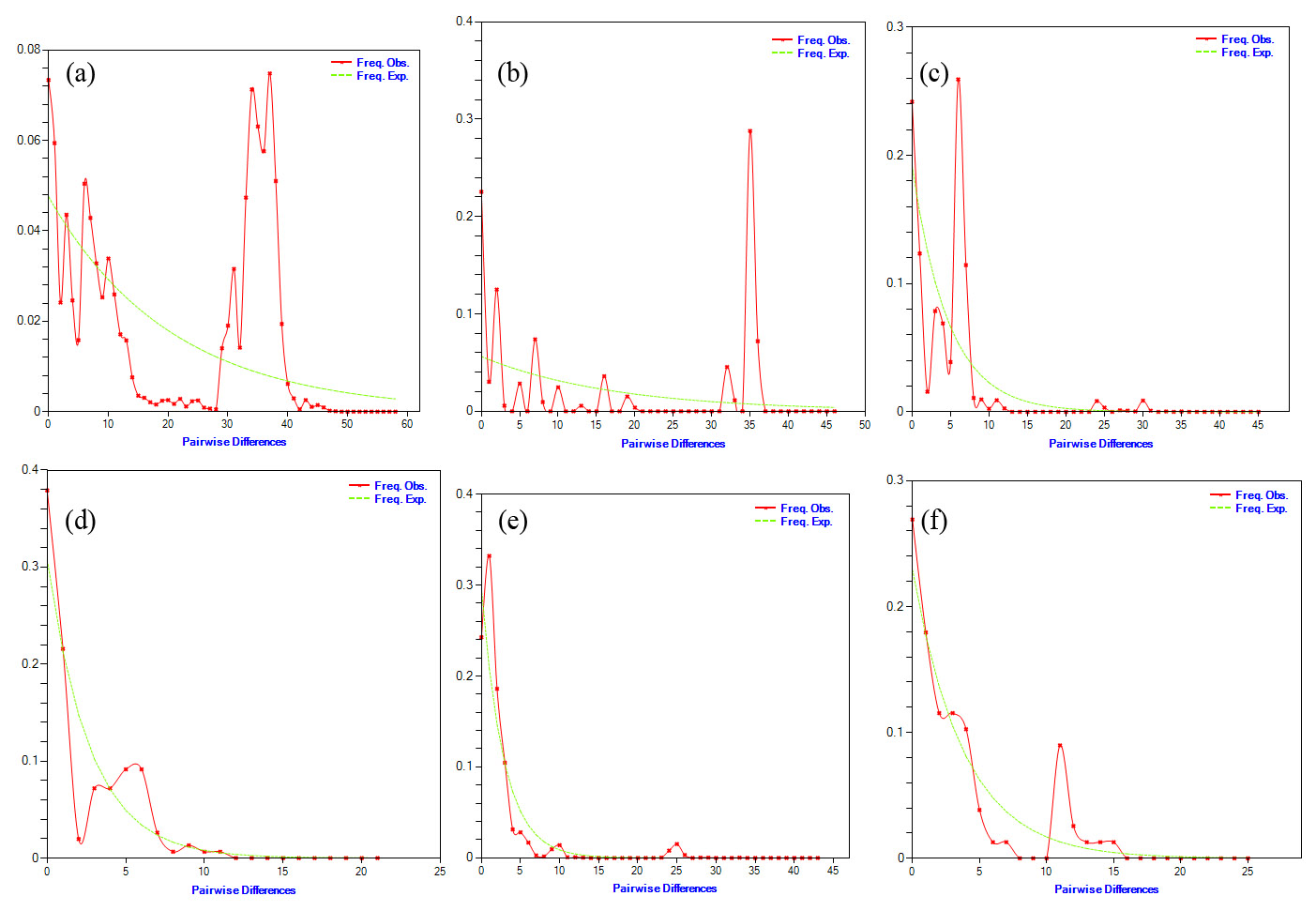

Supplement: Supplementary file 7 — Supplementary Material 7 [file 12870_2024_4783_MOESM7_ESM.jpg]

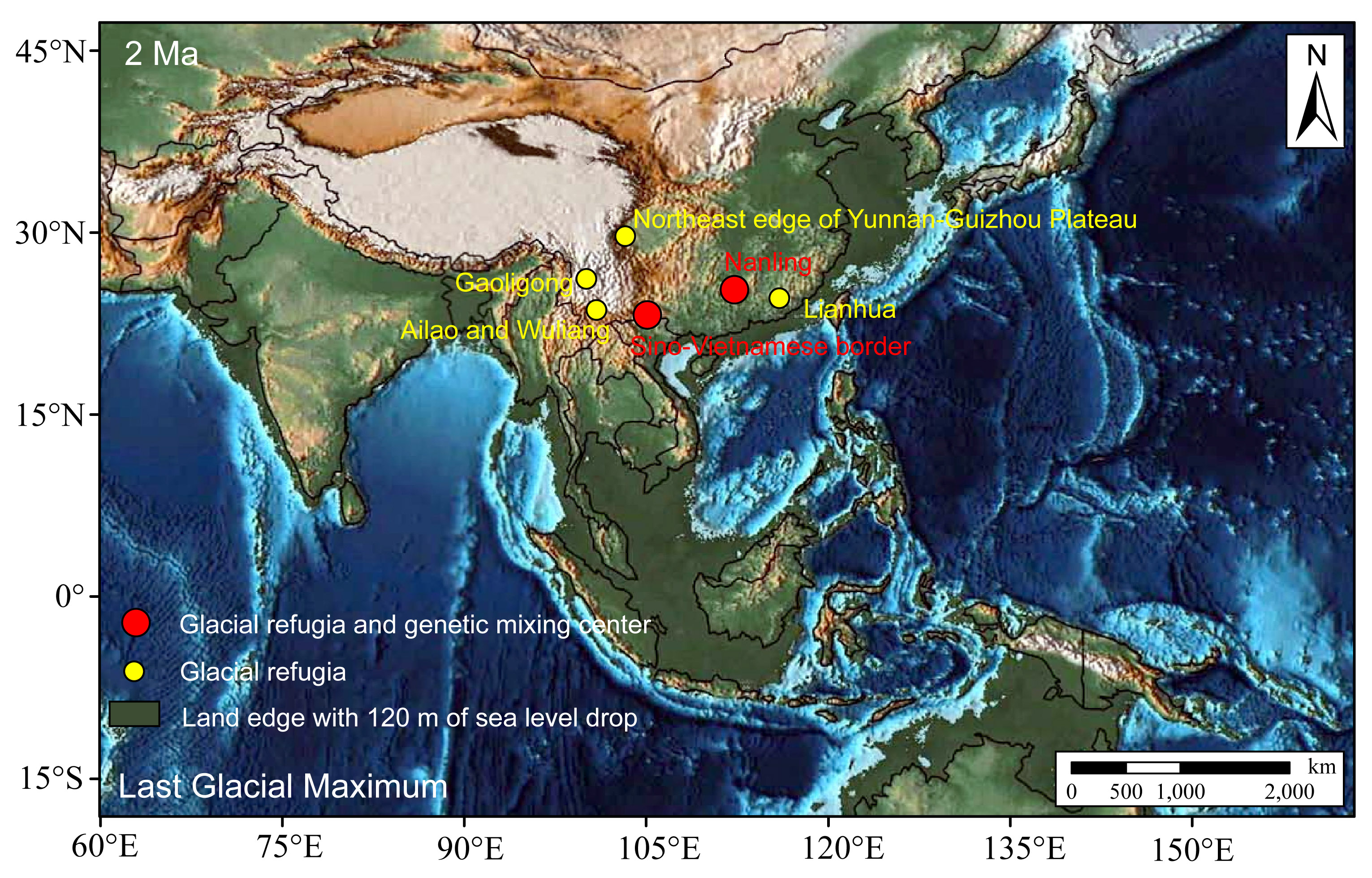

Supplement: Supplementary file 8 — Supplementary Material 8 [file 12870_2024_4783_MOESM8_ESM.jpg]
