# Supplementary material for: Phylogeographical and population genetics of Polyspora sweet in China provides insights into its phylogenetic evolution and subtropical dispersal
Source: BMC Plant Biol. 2024 Feb 6;24:89. doi: 10.1186/s12870-024-04783-5 (PMC10845455; doi:10.1186/s12870-024-04783-5)
Supplement: Supplementary file 9 — Supplementary Material 9 [file 12870_2024_4783_MOESM9_ESM.docx]

**Supplementary Figure Captions**

**Figure S1.** Phylogenetic tree of Theaceae based on chloroplast genomes by Maximum likelihood and Bayesian inference methods. The numbers preceding the slashes on the nodes indicate the support rates from the ML tree, while the numbers following the slashes represent the posterior probability values acquired from the BI tree. Asterisks represent nodes with maximal support, ML support rates of 100% and BI posterior probability values of 1.00. A: *Polyspora axillaris*, B: *P. chrysandra*, C: *P. hainanensis*, D: *P. longicarpa*, E: *P. tiantangensis*, F: *P. speciosa*, G: *P. tonkinensis*, H: *P. kwangsiensis*.

**Figure S2.** Phylogenetic tree of Theaceae based on ribosomal 18S-26S rRNA by ML/BI. The numbers preceding the slashes on the nodes indicate the support rates from the ML tree, while the numbers following the slashes represent the posterior probability values acquired from the BI tree. Asterisks represent nodes with maximal support, ML support rates of 100% and BI posterior probability values of 1.00.

**Figure S3****.** Phylogenetic tree of Theaceae by ML/BI methods constructed from the combined dataset of ribosomal 18S-26S rRNA and chloroplast genome (1 IR region removed). The numbers preceding the slashes on the nodes indicate the support rates from the ML tree, while the numbers following the slashes represent the posterior probability values acquired from the BI tree. Asterisks represent nodes with maximal support, ML support rates of 100% and BI posterior probability values of 1.00. A-H: *Camellia reticulata*，B-I: *Polyspora longicarpa*, C-J: *Pyrenaria spectabilis*, D-K: *Apterosperma oblata*, E-L: *Schima wallichii*, F-M: *Gordonia lasianthus*, G-N: *Stewartia sinensis*. Species name in red font represent Chinese *Polyspora* species.

**Figure S4.** Chronological tree of the differentiation of Theaceae species with five fossil calibrations and a maximum restricted age of 125 Ma. The numbers of the red nodes represent five fossil calibration points, as detailed in Table S1.

**Figure S5.** Interpopulation haplotype network based on combined cpDNA regions (*trn*H-*psb*A, *rpo*B-*trn*C, and *pet*N-*psb*M); Size of the circle was pro-portional to the relative frequency of the haplotype; Different color represents different populations.

**Figure S6.** Mantel test of genetic and geographic distance of *Polyspora* based on DNAsp. (a) *Polyspora*; (b)*P. speciosa*; (c) *P. axillaris*; (d)*P. chrysandra*.

**Figure S7.** Mismatch distribution analysis of *Polyspora* based onDNAsp, (a) *Polyspora*, (b) *P. axillaris*, (c) *P. speciosa*, (d) *P. longicarpa*, (e) *P. chrysandra*, (f) *P. hainanensis*

**Figure S8.** Glacial refugia and genetic mixing center of *Polyspora* in China. Source of base map data (Cao et al., 2017).

**References**

Cao W, Zahirovic S, Flament N, et al. Improving global paleogeography since the late Paleozoic using paleobiology[J]. Biogeosciences, 2017, 14(23): 5425-5439.
